# Who Migrates and Who Returns in a Context of Free Mobility? An Analysis of the Reason for Migration, Income and Family Trajectories

**DOI:** 10.1007/s10680-023-09667-2

**Published:** 2023-06-22

**Authors:** Rosa Weber, Jan Saarela

**Affiliations:** 1 Department of Sociology, Stockholm University, 106 91 Stockholm, Sweden; 2 Institut National d’Études Démographiques, 93300 Paris, France; 3 Åbo Akademi University, Postbox 311, 65101 Vaasa, Finland

**Keywords:** Reason for migration, Income trajectories, Trajectories of family formation, Return migration, Free mobility, Linked register data

## Abstract

The establishment of free mobility in Europe has lowered barriers to movement and given rise to diversity in migration and integration patterns. However, in part due to data constraints, it is difficult to study migration motives, integration and return migration together. Using linked Finnish and Swedish register data covering the period 1988–2005, we address these processes within the same framework and study how the reason for migration and trajectories at the destination relate to return migration. In particular, we assess the migration motives of 13,948 Finnish migrants in Sweden using pre- and post-migration information. Finland and Sweden have been part of the common Nordic labour market since 1954, which has allowed Nordic citizens to move without barriers between the two countries. We also study how income trajectories and trajectories of family formation differ across the assessed motives, and analyse how return migration risks are shaped by the motive and by trajectories of income and family formation. Results reveal that labour and tied migrants are initially more likely to have family abroad than student migrants. Student migrants instead continue their education and experience a steeper income increase. The income of student migrants eventually catches up and surpasses that of labour migrants. Return migration risks are shaped by trajectories at the destination, but also by the initial migration motive. These findings underline the importance of assessing diversity across migrants to gain a better understanding of how different migrant groups fare in the destination country and how this relates to subsequent moves.

## Introduction

As migration has become easier in the European Union (EU), the reasons for migration have become more diverse (Castro-Martín & Cortina, 2015; Verwiebe et al., 2014). This increased variety in migration motives has given rise to complex patterns of integration as well as return and circular migration (Kleinepier et al., 2015; Luthra et al., 2018). Considering that migrants are free to move without a residence or work permit in open border settings, official records do not collect information on migration motives or admission categories. The latter is often used to proxy migration motives in other contexts, as it roughly captures the legal framework along with barriers and support systems under which migrants enter the country (Bevelander, 2011; Luik et al., 2016; Ruiz & Vargas-Silva, 2017; Sarvimäki, 2017). Some studies have relied on the country of origin to assess motives, but in many instances, migrants from the same origin country have different migration motives (Burrell, 2010).

In contrast, survey data often include information on migrants’ self-reported reason for migration, but they generally cannot provide reliable estimates on return migration risks, which are considerable in open border settings (Engbersen et al., 2017; Favell, 2008, 2009). Longitudinal information on integration outcomes is also often limited in survey data. This information is relevant for assessing short- versus longer-term outcomes.

Prior research has shown that international student migrants constitute a growing proportion of the migrant population (King, 2018; Wit et al., 2013). Still, migration driven by economic reasons remains an important part of intra-EU migration (Becker & Teney, 2020; Bygnes & Erdal, 2017; Kleinepier et al., 2015). When it comes to integration, studies have shown that non-economic migrants who move within the EU tend to enter the labour market more slowly than economic migrants (Luthra et al., 2018). Research has also indicated that migrants’ time spent abroad has become more variable and decisions to stay, return migrate and engage in circular migration more complex (Drinkwater & Garapich, 2015; Engbersen et al., 2013, 2017). The literature to date has nevertheless focused on migration from East to West Europe, and in particular on Polish migration to the UK (Drinkwater & Garapich, 2015; Kilkey & Ryan, 2021; Kleinepier et al., 2015; Trevena, 2013), or on highly educated migrants such as physicians (Bartolini et al., 2017; Becker & Teney, 2020). However, contextual factors that are specific to the country of origin, destination and period may play an important part in shaping migration patterns. In order to disentangle context-specific patterns from more general migration patterns, it is important to expand the literature to other settings.

This study analyses the Finnish-Swedish migration context and examines how the reason for migration and trajectories at the destination relate to return migration. The Nordic setting provides insight into migration trends in a context of free mobility that has been in place for more than 50 years, which may be interpreted as a potential precursor to European migration. Using linked Finnish and Swedish register data that provide information on pre- and post-migration characteristics, we address four research aims. First, we approximate the reason for migration, distinguishing among labour, student and tied migrants, as well as a residual category in a data set where direct information on the reason for migration is not available. Second, we assess how income trajectories and trajectories of family formation differ by the reason for migration. Third, we analyse how return migration risks are shaped by the reason for migration as well as income trajectories and trajectories of family formation. Fourth, we study how the reason for migration relates to seasonal patterns and the likelihood of circular migration.

Linked Finnish and Swedish register data allow us to connect migrants’ main activities in the origin and destination country. Migration and integration are inherently linked and extend beyond the national context (King, 2002). Focusing only on one country thus provides a partial understanding of these dynamics. Yet, few data sets incorporate information from the origin and destination country. Some notable exceptions are survey data collected by the Mexican Migration Project (MMP), Latin American Migration Project (LAMP), the Migrations Between Africa and Europe Project (MAFE) and the Polish Migration Project (PMP). However, return and circular migration risks are difficult to assess without population-register data. Linked Finnish and Swedish population-register data used in this study are at the individual level and cover the years 1988–2005. This allows us to address the reason for migration, patterns of integration, and risks of return and circular migration during a relatively long follow-up period.

Disentangling the processes that drive migration and migrants’ situation in the destination country is generally intricate as the majority of migrants are young adults. In 2019, half of all migrants coming to the EU were under the age of 29 (Eurostat, 2020). During early adulthood, several parallel processes, such as migration, family and employment trajectories, occur within a relatively short time span and influence each other. This interaction makes it difficult to study one trajectory independently of another. A growing literature analyses these parallel processes (Sirniö et al., 2017; Vidal et al., 2020), and although studies increasingly attempt to incorporate international migration (Castro-Martín & Cortina, 2015; Wingens et al., 2011), there continues to be a need to embed migration in a life course perspective. The context of free mobility analysed in this study has prompted greater heterogeneity in the reason for migration and thus provides a novel setting in which we can study this interplay in an accentuated form.

In the next sections, we review the literature on migration decisions and discuss the Finnish-Swedish migration context analysed in the empirical part of the paper. Then, we describe our data and methods and present the empirical findings. We conclude with a summary of the results and a consideration of their theoretical implications.

## Theoretical Background and Previous Literature

Current migration within Europe is shaped by a diverse set of reasons (Verwiebe et al., 2014). While economic and family migration constitute an important part of intra-EU movement (Becker & Teney, 2020), they are supplemented by other migration forms, such as student and retirement migration (Börjesson, 2017; Klinthäll, 2006).

This section provides a review of the previous theoretical and empirical literature. Rather than providing an in-depth account of one theory, or an exhaustive discussion of different migration theories, we focus on three theories—the human capital model, social network theory and global youth mobility cultures—in order to address different migration and integration processes within the same framework.

### Reason for Migration

Labour migrants’ primary motive in moving is work-related. According to the *human capital model*, individuals are expected to relocate if the gains exceed the cost of migration (Harris & Todaro, 1970; Sjaastad, 1962). In light of cross-country differences in the returns to skills, individuals migrate if the returns to their skill set are higher abroad (Borjas & Bratsberg, 1996). Unemployed individuals may have particularly high incentives to move if they have better prospects to enter the labour market in the destination than the origin country. However, models that solely focus on economic factors have been criticised for providing a simplistic account of migration decisions (Arango, 2004). *Social network theory* argues that non-migrants draw on social capital embedded in connections to migrants (MacDonald & MacDonald, 1964; Massey et al., 1998). These connections lower the uncertainties involved in migration, as prior migrants pass on information about opportunities in the destination country. Previous work shows that this kind of information plays an important role in stimulating labour migration (Kalter, 2011; Munshi, 2003).

Student migrants move in order to acquire human capital abroad. In other words, student migration is a career-enhancing investment as hypothesised by the *human capital model* (King & Sondhi, 2018). *Social network theory* is also relevant when discussing student migration, considering that many have personal or family ties to other countries (Waters & Brooks, 2010). A third conceptual frame considered in the literature refers to *global youth mobility cultures*, where the freedom to travel constitutes an important part of students’ experience (King & Sondhi, 2018). Indeed, student migrants often explain their decision to study abroad with ideas about gaining intercultural awareness and seeking adventure, besides noting academic and career advancement (Findlay et al., 2017; King & Ruiz‐Gelices, 2003).

Even though migration theories have focused on the individual level, migrants often move with their partner or family (Kofman, 2004). Theoretically, this issue was first highlighted by Mincer (1987) in an expansion of the *human capital model*. He argued that partners migrate if both expect to gain from migration, but they may also migrate if the gains made by one partner are high enough to motivate relocation. In that case, one partner is the lead migrant, whereas the other is the tied migrant, who joins or follows. Tied migrants thus often prioritise relocating with their partner over finding the optimal destination for their career progression (Brandén, 2014; Brandén & Haandrikman, 2018). According to *social network theory*, having connections in the destination country generally helps migrants find work. However, tied migrants may also experience less pressure to enter employment to support themselves (Boyd & Nowak, 2012).

### Income Trajectories and Trajectories of Family Formation

The migration motive is important in guiding predictions about both migration flows and migrants’ incorporation into the destination country (Kanas & Steinmetz, 2021; Zwysen, 2019). Thus, labour, student, and tied migrants are expected to follow distinct income trajectories. Labour migrants move for work, and the *human capital model* leads us to expect that they choose the destination and timing accordingly (Harris & Todaro, 1970; Massey et al., 1993; Sjaastad, 1962). Their income can therefore be assumed to be higher than that of other migrant groups in the first years after immigration.

In contrast, non-economic migrants move to pursue their studies or to be with family. Assuming that student migration is a career-enhancing investment, as the *human capital model* posits, student migrants’ income is likely lower than that of the other migrant groups while they attend university or any other school (King & Sondhi, 2018). However, if the investment pays off, student migrants have good chances of getting a well-qualified position when they have completed their studies (Maskileyson et al., 2021). Considering that many student migrants return after finishing their studies, those who stay are likely positively selected on skills and intrinsic motivation (King et al., 2010; Mosneaga & Winther, 2013). After some years in the destination country, student migrants’ income can therefore be expected to exceed that of the other migrant groups.

Prior research also shows that tied migrants are more often unemployed or outside of the labour force in the destination country, when compared to lead migrants (Krieger, 2020; Le, 2003; Taylor, 2007). This may be due to responsibilities to take care of children or other family members. Although tied migrants are expected to take longer to locate work than labour migrants, their income may exceed that of student migrants while the latter group is studying.

Regarding trajectories of family formation, tied migrants are expected to be more likely to have family abroad, that is to be married or a parent, than other migrant groups, as they move with their partner (Andersson et al., 2015). Similarly, having a stable income may provide labour migrants with some security to start a family. In contrast, student migrants may be less prone to make long-term plans to stay and form a family in the destination country, as hypothesised by *global youth mobility cultures* (Findlay & King, 2010).

### Return and Circular Migration

Some migrants return as part of a planned strategy, while others decide to return after having spent some time in the destination country. According to the *human capital model*, stable employment makes it more beneficial to stay in the destination country (Constant & Zimmermann, 2011, 2012). Still, the model considers a number of reasons why labour migrants may return migrate and engage in circular migration. First, unmet expectations, i.e. a negative shock, can induce labour migrants to return. For instance, labour migrants often return if they do not find a job or earn lower wages than they expected (Borjas & Bratsberg, 1996; Dustmann, 1996; Rooth & Saarela, 2007). Second, based on *social network theory*, labour migrants may have a strong incentive to return if they left their family behind. This can also prompt circular migration (Engbersen et al., 2013).

When it comes to student migrants, the *human capital model* argues that they return migrate after completing the programme or obtaining study credits if they expect higher returns on their career-enhancing investment in the origin than the destination country. Otherwise, they might stay in the destination country (Mosneaga & Winther, 2013). Prior research also shows that going abroad as an Erasmus student is strongly associated with the subsequent likelihood of working in a foreign country among German and UK students (Findlay et al., 2017; Parey & Waldinger, 2011).

In contrast, having a family tends to constrain migration, as a spouse and children make it more difficult to realise a move, i.e. make it more costly to relocate, according to the *human capital model* (Dustmann, 2003). Tied migrants can therefore be expected to have moderate return and circular migration risks.

## Migration Context

Finnish migrants constitute the third largest immigrant group in Sweden today, after Syrians and Iraqis. They account for about 140,000 persons (Statistics Sweden, 2022). This high number is mainly the result of the large migration flow from Finland to Sweden in the decades following World War II (Hedberg & Kepsu, 2003; Korkiasaari, 2003). Labour migration from Finland to Sweden peaked in the early 1970s and has continued at a lower rate since then. During our study period, 1988–2005, migration rates between the two countries were modest. Both Finland and Sweden were hit by the economic recession in the early 1990s. Still, considering the long history of labour migration between the two countries, many Finns had a large network in Sweden, which presumably increased their likelihood of moving. Some also continued to move to Sweden to improve their economic position, but the gains made by moving were not high enough to create a strong incentive to move to Sweden for higher life-time earnings. In contrast, student migration as well as Erasmus migration from Finland increased over this period (King et al., 2010). Indeed, migration to Sweden often corresponds to leaving the parental home (Hedberg & Kepsu, 2003). Finnish migrants who moved to Sweden are the focus of the empirical analysis. The reverse flow, of Swedes to Finland, has been consistently small.

Sweden and Finland are geographically, culturally and historically close. The labour market structure, educational system and social benefits are also similar in the two countries, indicating that barriers to migration are low (Saarela & Scott, 2019). Even though the main languages spoken in the two countries differ considerably, a minority of Finns grow up speaking Swedish. About five per cent of the Finnish population has Swedish as their registered mother tongue in the population register. Previous research reveals considerable differences in migration and integration patterns of Finnish and Swedish speakers (Saarela & Scott, 2017; Weber & Saarela, 2019).

## Data

Our data set was constructed by integrating records of Finnish immigrants in Sweden from population registers in both Sweden and Finland.^1^ The two data sets were linked by the identification of migrants based on their unique personal identity numbers (PINs). Linkage was fully successful, but since Statistics Finland had a policy of not providing data on total populations, the data at hand constitute a 77.5% random sample. Through the linkage we have detailed information on pre- and post-migration characteristics. We measure migration by registration and deregistration from the population registers in each country. Nordic citizens, who move between the Nordic countries, are required to register a move if they intend to stay abroad for more than six months (FPA, 2022; Swedish Tax Agency, 2022). Most register even shorter sojourns, as there are high incentives to do so. For instance, one needs a PIN to open a bank account, rent a flat, or to receive income. We can thus identify migrants who move back and forth between Finland and Sweden and assess the reliability of these records by verifying that migrants who deregister in Finland appear in the Swedish register, and vice versa. Comparing the month of exit from Finland and entry in Sweden, we find that for 98% of all moves, the timing of the migration in each country’s register differs by less than two months.

The raw data from Sweden cover the period 1985–2005 and contain rich information on socioeconomic, demographic and labour market characteristics of individuals who migrated to Sweden. The raw data from Finland cover the years 1987–2007 and contain information on similar variables of the same persons, who are linked to the Swedish registers.

### Data Restrictions

We make a number of data restrictions. First, we focus on Finnish-born individuals who made their first move to Sweden between 1988 and 2004. This ensures that we have information from both countries and observe individuals for at least one year after immigration. Records in the Swedish register data on each individual’s previous moves (to and from Sweden) allow us to establish the first move of each migrant, even if it occurred before 1985. We use this information to exclude persons who experienced a move from Finland to Sweden prior to 1988, in order to avoid problems of left truncation. Second, we exclude individuals who migrate and return migrate in the same year, as we cannot analyse their income trajectories and trajectories of family formation. In order to identify the reason for migration, we build on information from the Finnish register recorded in the calendar year before the move and information from the Swedish register recorded in the calendar year of the move. To circumvent tautological problems in which information from the year of arrival in Sweden would be used in both the categorisation and the analysis, our analyses start observing migrants the calendar year after the year of arrival (henceforth referred to as the year after arrival). This ensures that our categorisation is distinct from the patterns observed in the analysis. Third, we focus on individuals aged 19 to 30 at immigration. The lower age limit is 19 in order to include information on the matriculation examination, which serves as a prerequisite for entrance into university studies and is usually obtained at this age. The upper age limit is 30, as most people have finished their education, entered the labour market and potentially started to form a family by then. Moreover, more than two-thirds of moves between Finland and Sweden occur between the ages of 19 and 30. In this way, we analyse the bulk of movement while simultaneously focusing on an age group where labour market participation, studies and family formation are common alternatives. After these data restrictions, the final sample comprises 13,948 migrants from the cohorts born 1958–1985.

### Identifying the Reason for Migration

In order to address our first research aim, i.e. to approximate the reason for migration, we build on information from the origin and destination country in Finnish and Swedish register data. This provides us with relatively good insight into migrants’ situation prior to and following the move and allows us to identify the reason for migration among Nordic citizens who move between these two Nordic countries, even though there are no official records on the reason for migration. When approximating the reason for migration, we differentiate between three major categories: labour, student and tied migrants. Although this does not capture the complete range of motivations to migrate, we are still able to gain an understanding of heterogeneity within the total migrant group by focusing on reasons that have been argued to be very common (Becker & Teney, 2020).

Considering that *labour migrants* move abroad for work, we build on information about individuals’ employment status in Sweden in the year of arrival. The employment status is recorded in November of each calendar year in Swedish register data. To identify an individual’s employment status, Statistics Sweden relies on control statements that employers are required to send to the tax authority and tax declarations provided by the self-employed. This information is compiled in November, which serves as a reference month for Statistics Sweden when assessing whether individuals hold a job or not (Statistics Sweden, 2016).

**Fig. 1:**
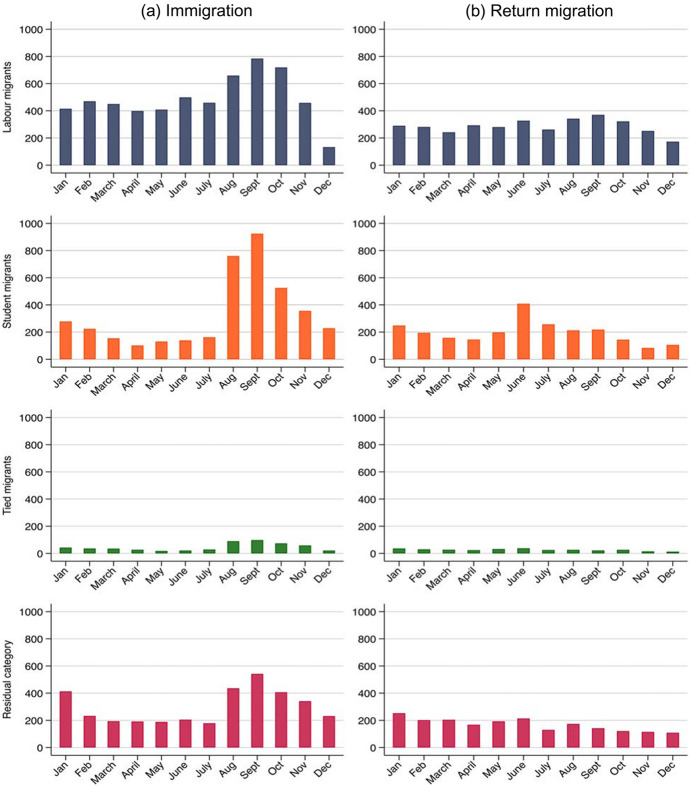
Number of immigrants and return migrants by month of migration and reason for migration. *Note* The x-axis refers to the number of individuals

Migrants who moved in November and December may be misclassified because they have not had enough time to find employment in the year of immigration. We have assessed whether this is a serious concern by observing the share of individuals who were recorded as employed (in November) by the month of arrival, and compared this to the share of individuals with nonzero (annual) income in the year of immigration. We found that this is only a problem for migrants who arrived in December and ran additional analyses dropping observations for December movers. However, because of the relatively small number of immigrants in this month (four percent of all immigrants; see also Fig. 1), this resulted in a similar classification of the reason for migration.

Finnish registers additionally provide us with information on individuals’ main activity in the year prior to migration, which we can use to distinguish between persons who were employed, unemployed, studying, in the army service, or outside of the labour force. Our categorisation identifies labour migrants as individuals who were employed, unemployed, in the army service, or studying in Finland the year before the move, and who were employed in Sweden in the year of the move (N = 5853).

*Student migrants*, by contrast, move abroad to study. Seeing that the matriculation examination is not only a prerequisite for entering university studies in Finland, but also often used as an eligibility requirement for university studies in Sweden, we identify student migrants as individuals who have passed the matriculation examination in Finland prior to migration, and who were non-employed in Sweden in the year of migration (N = 3981). Considering that some students work while studying at university, our categorisation makes no restrictions regarding the employment status after the year of arrival. Moreover, some student migrants move for a full degree, while others are study-credit movers. Therefore, we make no restrictions regarding whether they have completed a degree.

*Tied migrants* are individuals whose primary motive is to follow or join their partner. The detailed information provided in both registers allows us to observe individuals moving in the same year and month, from the same region in Finland, to the same municipality in Sweden, and with the same position in the household in the year prior to migration. The position in the household distinguishes between individuals who were married with children, married without children, co-habiting with children, co-habiting without children, or none of the above, i.e. individuals who lived in a single household, with their parents, or were single parents prior to migration. We focus on individuals who move in pairs and are of opposite sexes. Moreover, we assume that tied migrants move with a labour or student migrant. In other words, we do not assign individuals the status of tied migrant if two migrants move together but neither is categorised as a labour or student migrant. Conversely, if two individuals of opposite sex move at the same time but both are classified as labour or student migrants, neither is identified as a tied migrant. Using information on households from the Swedish register, based on individuals’ marital and shared parenthood status, we additionally identify migrants who live with their partner in Sweden in the year of immigration. This strategy leaves us with 551 tied migrants. An assessment of whom tied migrants follow reveals that 48% of tied migrants move with a labour migrant, 41% move with a student migrant, and 11% join their partner in Sweden.

There are a number of individuals who do not fall into any of the three major categories. For the sake of completeness, these are assigned to a *residual category* that includes a broad range of activities in Finland and Sweden (N = 3563).

### Descriptive Statistics

In order to appraise our classification, we present pre-migration characteristics of labour, student, tied migrants and migrants in the residual category. Table 1 shows that 56% of the migrants were female, but women were especially overrepresented among student migrants (70%). Student migrants were also generally younger in the year before the move than other migrants (about half were age 19–22). Swedish speakers were overrepresented among all migrant groups, but especially so among student and tied migrants. One in three migrants lived in a single household prior to migration, and nearly two in three student migrants moved out of their parental home. Regarding individuals’ main activity, we find that most migrants were employed in the year prior to migration (44%). Still, labour migrants were the most likely group to be employed (56%). Among student migrants, about 40% studied in the year before the move, whereas tied migrants and migrants in the residual category were often outside the labour force. Tied migrants and migrants in the residual category generally had a lower educational attainment before migration than the other groups. Among labour migrants, nearly one in three had completed tertiary education prior to migration. Few migrants were married before migration (9%). However, tied migrants were more likely to be married than the other groups, with 17% being married prior to migration. About 40% of migrants lived in Southern Finland the year before the move, but moving out of this region was most common among student migrants (47%).

**Table 1:** Distribution of pre-migration characteristics by migrant type (proportion within groups reported, except for the median year of immigration)

	Labour migrants	Student migrants	Tied migrants	Residual category	All migrants
*Demographic characteristics*					
Female	0.52	0.70	0.44	0.48	0.56
Age at immigration: 19–22 years	0.31	0.49	0.45	0.43	0.39
Age at immigration: 23–26 years	0.42	0.34	0.33	0.35	0.38
Age at immigration: 27–30 years	0.27	0.17	0.22	0.22	0.23
Swedish speaker	0.32	0.35	0.37	0.34	0.34
*Position in the household*					
Living in a single household	0.28	0.25	0.23	0.32	0.28
Living in parental home	0.49	0.59	0.50	0.48	0.52
Single parent	0.01	0.01	0.02	0.03	0.01
Cohabitant without children	0.12	0.09	0.07	0.08	0.10
Cohabitant with children	0.01	0.01	0.04	0.03	0.02
Married without children	0.04	0.03	0.04	0.01	0.03
Married with children	0.04	0.03	0.11	0.04	0.04
*Main activity*					
Employed	0.56	0.36	0.36	0.33	0.44
Unemployed	0.12	0.06	0.09	0.12	0.10
Studying	0.29	0.42	0.17	0.15	0.29
Army service	0.03	0.03	0.03	0.02	0.03
Other outside the labour force	N.A	0.12	0.34	0.39	0.15
*Education*					
Primary educ. (< 11 years)	0.16	N.A	0.41	0.47	0.20
Secondary educ. (11–12 years)	0.57	0.84	0.47	0.44	0.61
Tertiary educ. (13–17 years)	0.27	0.16	0.12	0.09	0.18
*Marital status*					
Unmarried	0.90	0.91	0.82	0.91	0.90
Married	0.09	0.08	0.17	0.07	0.09
Divorced	0.01	0.01	0.01	0.02	0.01
*Region of residence*					
Southern Finland	0.41	0.47	0.35	0.28	0.39
Western Finland	0.35	0.30	0.28	0.23	0.30
Eastern Finland	0.05	0.04	0.03	0.04	0.04
Oulu	0.07	0.04	0.05	0.05	0.05
Lapland	0.07	0.06	0.16	0.12	0.08
Åland Islands	0.05	0.08	0.11	0.18	0.09
Unknown	N.A	0.01	0.03	0.09	0.03
*Birth cohort*					
1958–1965	0.26	0.10	0.16	0.21	0.20
1966–1970	0.36	0.21	0.23	0.28	0.29
1971–1975	0.20	0.25	0.23	0.19	0.21
1976–1980	0.15	0.30	0.24	0.20	0.21
1981–1985	0.04	0.15	0.13	0.13	0.10
Median year of immigration	1992	1998	1998	1994	1995
Number of migrants	5853	3981	551	3563	13,948
Number of return migrants	3434	2372	314	2025	8145

Pre-migration characteristics are measured one year before migration from Finland

## Methods

Based on the approximated reason for migration, we begin by studying the seasonality in immigration and return migration. Thereafter, and as described in greater detail below, we analyse migrants’ income trajectories and trajectories of family formation and assess how the risk of return migration is shaped by the reason for migration and trajectories in the destination country. As a final exercise, we study how the reason for migration relates to seasonal patterns and the likelihood of circular migration. Due to the relatively small number of circular migrants, this final part is more cursory.

### Income Trajectories and Trajectories of Family Formation

**Fig. 2:**
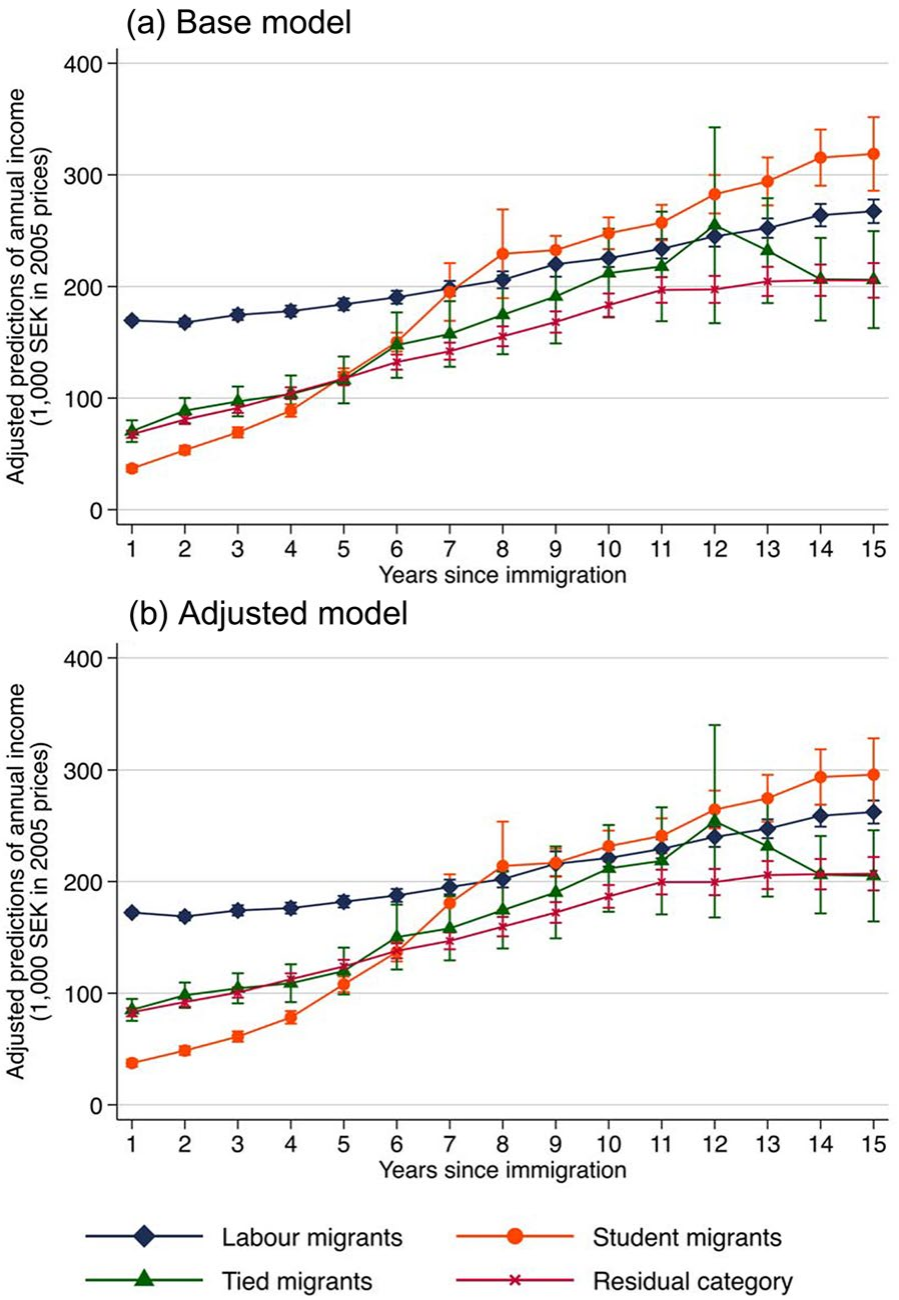
Adjusted predictions (with 95% confidence intervals) from ordinary least squares regressions estimating income trajectories from the reason for migration, years since migration and selected control variables. *Note* These numbers are based on Eq. (1), which includes an interaction term between the reason for migration and years since migration. In the base model (panel a), control variables include gender, age at immigration, being a Swedish speaker and year of immigration. In the adjusted model (panel b), educational attainment is additionally controlled for as a time-varying variable. Standard errors are clustered at the individual level. Corresponding results are provided in Table S1 in the Supplement

Income trajectories are captured using information on annual earnings in Sweden, which is recorded at the end of each calendar year. We follow migrants for 15 years, starting the year after arrival. Migrants are right-censored when they return, move to a third country, die, at the end of the observation period in 2005, or after 15 years in Sweden, whichever comes first. Income is inflation adjusted to 2005 prices. We specify the base model:

1 $$Y_{it} = \alpha + \beta {\text{reason}}\,{\text{mig}}_{i} + \delta {\text{years}}\,{\text{since}}\,{\text{mig}}_{it} + \mu \left( {{\text{reason}}\;{\text{mig}}_{i} \times {\text{years}}\;{\text{since}}\;{\text{mig}}_{it} } \right) + \gamma X_{i} +\epsilon_{it } ,$$

where $Y_{it}$ refers to annual income in Sweden for migrant *i* in year *t*. The outcome variable is thus continuous, and we estimate linear regressions with ordinary least squares. The intercept is referred to as *α*. The term $\it {\text{reason }}\,{\text{mig}}_{i}$ is a set of dummy variables that indicates whether migrant *i* is a student, tied migrant or belongs to the residual category. Labour migrants represent the omitted reference category. $\beta$ represents the corresponding coefficient vector, which gives the average difference in income between each migrant group and the reference category (labour migrants). The term $\it {\text{years }}\;{\text{since }}\,{\text{mig}}_{it}$ is a set of dummy variables that indicates the number of years elapsed since migration, ranging from two to 15. The (omitted) reference category is one year since migration. This allows us to assess any nonlinear relation between years since migration and income. The corresponding coefficient vector, $\delta$, provides the difference in income, in each year since migration *t*, compared to the reference year (one year since migration). We also include interaction terms between reason for migration and years since migration. This allows for separate income trajectories for each migrant group. $\mu$ refers to the coefficient vector for all possible combinations between the two sets of dummies (in total 42 coefficients, i.e. 14 per migrant group). $X_{i}$ is a set of time-constant control variables–gender, age at immigration, being a Swedish speaker and year of immigration–and *γ* is the corresponding coefficient vector.^2^$\epsilon_{it }$ represents the error term. Standard errors are clustered at the individual level, as we analyse one observation per calendar year. We present adjusted predictions, which provide the estimated mean annual income over time since migration for each migrant group, when other variables are set to their means (see Fig. 2).^3^ We also estimate an adjusted model that additionally includes educational attainment as a time-varying variable. This allows us to assess whether the attainment of an educational degree in Sweden underlies part of the income trajectories.

**Fig. 3:**
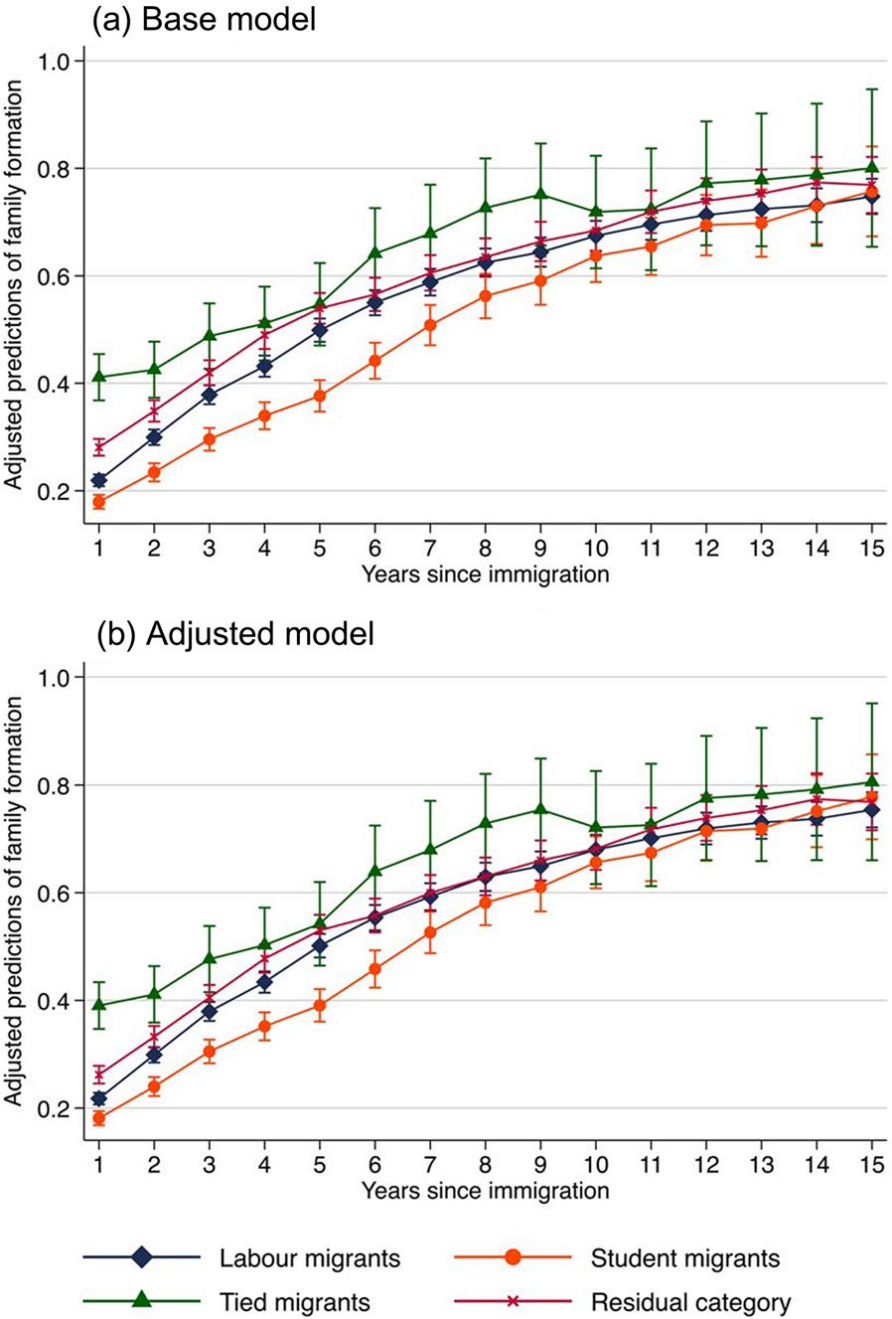
Adjusted predictions (with 95% confidence intervals) from logistic regressions estimating trajectories of family formation (being married or a parent) from the reason for migration, years since migration and selected control variables. *Note* These numbers are based on Eq. (2), which includes an interaction term between the reason for migration and years since migration. In the base model (panel a), control variables include gender, age at immigration, being a Swedish speaker and year of immigration. In the adjusted model (panel b), educational attainment is additionally controlled for as a time-varying variable. Standard errors are clustered at the individual level. Corresponding results are provided in Table S2 in the Supplement

Trajectories of family formation are captured using a time-varying indicator that is equal to one in the year when an individual has entered the first marriage or parenthood and in the subsequent years, and zero in the years prior to family formation.^4^ Swedish register data do not provide information on cohabitation without children. We are therefore restricted to measuring family formation based on marriage and parenthood. We observe migrants for 15 years, starting the year after their arrival in Sweden, and capture their trajectories of family formation in Sweden. Some migrants have married or entered parenthood prior to immigration. In such cases, the indicator variable is equal to one throughout the observation window. Since the outcome variable is binary, we estimate logistic regressions. The base model is:

2 $${\text{logit }}\theta_{it} = \ln \left( {\frac{{\theta_{it} }}{{1 - \theta_{it} }}} \right) = \alpha + \beta {\text{reason }}\;{\text{mig}}_{i} + \delta {\text{years}}\;{\text{ since}}\;{\text{ mig}}_{it} + \mu \left( {{\text{reason}}\;{\text{ mig}}_{i} \times {\text{years}}\;{\text{ since }}\;{\text{mig}}_{it} } \right) + \gamma X_{i} ,$$

where $\theta_{it}$ refers to the probability that migrant *i* enters the first marriage or parenthood in year *t* or has done so prior to year *t*. The set-up is otherwise similar to that described for analysing income trajectories above. We present adjusted predictions from this regression, which give the estimated mean probability over time since migration for each migrant group, when other variables are set to their means (see Fig. 3).^5^ Similar to the analyses of income trajectories, we also estimate an adjusted model that additionally includes educational attainment as a time-varying variable.

### Return Migration

We further analyse how the risk of return migration is shaped by the reason for migration and migrants’ situation in the destination country. Migration motives can structure both migrants’ situation in the destination country and may affect the return migration risk. Similarly, migrants’ situation over time in the destination country relates to the risk of return migration. Return migration, in turn, impacts the trajectories of labour, student and tied migrants in the destination country, as some groups, which experience high levels of return migration, become increasingly selected in the destination country. These factors are difficult to disentangle. By studying the processes within the same framework, we aim to gain an understanding of how they are related.

We estimate piecewise constant exponential models for the risk of return migration. These models allow the baseline hazard of return migration to vary over time spent abroad (Blossfeld et al., 2019). Persons enter the risk set (i.e. the observation window) in the year after arrival in Sweden. As previously mentioned, this is because our categorisation builds on information from the year of immigration to Sweden. We follow migrants for 15 years in Sweden and capture the timing and likelihood of return migration to Finland. Right-censoring is handled as described above. We specify the model:

3 $$\lambda_{i} \left( t \right) = \lambda_{0} \left( t \right) \times \exp \left\{ { \beta {\text{reason }}\,{\text{mig}}_{i} + \zeta {\text{educ}}_{it} + \eta {\text{income}}_{it} + \sigma {\text{family}}_{it} + \gamma X_{i} } \right\},$$

where $$\lambda_{i} \left( t \right)$$ refers to the risk to return migrate at time $$t$$. In this model, $$t$$ refers to the time since migration. The term $$\lambda_{0} \left( t \right)$$ indicates the baseline hazard, and $$\lambda_{o} \left( t \right) = \lambda_{j}$$ for $$t$$ in each interval $$[\tau_{J - 1} ,\tau_{J} )$$. We partition duration into 14 intervals with cut-points at the change of each calendar year. The term $$\it {\text{reason }}\,\,\,{\text{mig}}_{i}$$ is specified in the same way as above. Here, we also account for a set of time-varying indicators. These include a set of dummies for educational attainment ($$educ_{it} )$$, income quartile $$(income_{it}$$), and a dummy variable equal to one if individual *i* is married or a parent ($$family_{it} )$$. $$\zeta$$, $$\eta$$, and $$\sigma$$ represent their coefficient vectors. Similar to the equations above, $$X_{i}$$ represents time-constant control variables.

## Empirical Findings

### Seasonal Patterns in Immigration and Return Migration

Figure 1 reveals that the seasonality in migration is most pronounced among student migrants. Student migrants predominantly immigrate in August and September, which coincides with the start of the academic year. Return migration among student migrants is most common in June, which is when the academic year ends. Labour migrants are somewhat more likely to immigrate in August, September and October. This may be due to the fact that many employment contracts start in the autumn (Swedish Public Employment Service, 2020). Labour migrants’ likelihood of returning does not seem to be seasonal. Among tied migrants and migrants in the residual category, immigration is somewhat more common in August, September and October as compared with other months. Return migration does not appear to be seasonal in either of these two migrant groups, as we would expect.

### Income Trajectories and Trajectories of Family Formation

Income trajectories over time in Sweden provide insight into migrants’ economic situation in the destination country. Adjusted predictions of annual income are presented in Fig. 2. The base model (panel a) shows that labour migrants tend to have a higher income than the other migrant groups during the first years in the destination country. Even though differences between the other three groups are smaller, tied migrants and migrants in the residual category initially have a higher income than student migrants. However, student migrants have a steep earnings curve. The increase is particularly pronounced during the first eight years in the country. Subsequently, it flattens out and remains above 200,000 SEK. After about five years in the destination country, student migrants’ income exceeds that of tied migrants and migrants in the residual category. At the end of follow-up, student migrants’ income even surpasses that of labour migrants. When compared to labour migrants, migrants in the residual category have a lower income throughout the 15-year period. By contrast, the income of tied migrants comes close to that of labour migrants. However, tied migrants are a relatively small group and fluctuations should be interpreted with care. When we control for educational attainment in Sweden in the adjusted model (panel b), we find that the gap between student migrants and the other migrant groups becomes somewhat smaller. This suggests that attaining a Swedish degree is part of the explanation of why student migrants have a steeper earnings curve.

Figure 3 provides adjusted predictions of the likelihood of being married or a parent. The base model (panel a) shows that, in the first year after immigration, tied migrants are more likely to be married or to have children than the other groups. In contrast, student migrants are the least likely group to have a family in the first year after immigration. The proportion of student migrants who are married or parents remains lower over time in the destination country than in the other migrant groups, though differences between the groups become minimal after about eight years in the country. The patterns observed among labour migrants and migrants in the residual category fall between those of tied and student migrants. Differences between the base model (panel a) and the adjusted model (panel b) are small, which suggests that obtaining an educational degree in Sweden does not substantially change migrants’ trajectories of family formation, as we would expect.

### Return Migration

Table 2 provides hazard ratios from piecewise constant exponential models for the risk of return migration. Covariates are introduced in a stepwise fashion to show how estimates change when additional variables are included. The first model reveals that student migrants are more likely to return migrate than labour migrants. The risk of return migration of tied migrants and migrants in the residual category is similar to that of labour migrants. When we adjust for migrants’ demographic characteristics in Model 2, the difference between student and labour migrants becomes even larger. Still, it points to a moderate difference in relation to migrants’ demographic characteristics. Male migrants and Finnish speakers are almost twice as likely to return migrate than female migrants and Swedish speakers. Age at immigration is negatively related to the likelihood of return migration. Model 3 adds a time-varying indicator for educational attainment and shows that migrants with secondary education are less likely to return migrate than those with primary education. The effects for the other variables remain largely the same. However, when we include income in Sweden in Model 4, the hazard ratios for the reason for migration change considerably. At similar levels of income, tied migrants and migrants in the residual category have a lower risk of return migrating than labour migrants, while student migrants have a similar likelihood of return migrating. Women, older migrants and Swedish speakers are still less likely to return migrate than men, younger migrants and Finnish speakers. The relationship between education and the risk of return migration becomes non-significant. All else being equal, higher income is related to a lower risk of return migration. Migrants in the bottom income quartile are about twice as likely to return migrate than their counterparts in the top income quartile. The last model adds a time-varying indicator of marriage and parenthood and shows that migrants who are married or parents are less likely to return migrate, although the estimate is not significant.

**Table 2:** Hazard ratios from piecewise exponential models estimating the risk of return migration from the reason for migration and selected control variables

	(1)			(2)			(3)			(4)				(5)	
*Reason for migration*															
Labour migrants (ref.)	1			1			1			1			1		
Student migrants	1.14	***	(0.03)	1.25	***	(0.04)	1.26	***	(0.04)	0.95		(0.03)	0.95		(0.03)
Tied migrants	1.07		(0.06)	1.05		(0.06)	1.04		(0.06)	0.87	***	(0.05)	0.87	*	(0.05)
Residual category	1.01		(0.03)	0.98		(0.03)	0.97		(0.03)	0.81	***	(0.03)	0.81	***	(0.03)
*Demographic characteristics*															
Male (ref.)				1			1			1			1		
Female				0.68	***	(0.02)	0.68	***	(0.02)	0.67	***	(0.02)	0.67	***	(0.02)
Age at immigration (ref. 19–22 years)				1			1			1			1		
23–26 years				0.85	***	(0.02)	0.85	***	(0.02)	0.91	***	(0.02)	0.91	***	(0.02)
27–30 years				0.78	***	(0.02)	0.79	***	(0.02)	0.85	***	(0.03)	0.85	***	(0.03)
Finnish speaker (ref.)				1			1			1			1		
Swedish speaker				0.57	***	(0.01)	0.57	***	(0.01)	0.58	***	(0.01)	0.58	***	(0.01)
*Education*															
Primary educ. (ref.)							1			1			1		
Secondary educ							0.93	*	(0.03)	0.99		(0.03)	0.99		(0.03)
Tertiary educ							0.94		(0.04)	1.04		(0.04)	1.04		(0.04)
*Income quartiles* (ref. Quartile 1)										1			1		
Quartile 2										0.79	***	(0.02)	0.79	***	(0.02)
Quartile 3										0.54	***	(0.02)	0.54	***	(0.02)
Quartile 4 (top)										0.51	***	(0.02)	0.51	***	(0.02)
Not married and/or parent (ref.)													1		
Married and/or parent													0.97		(0.03)
Year of immigration (set of dummies)	No			Yes			Yes			Yes			Yes		
Split baseline hazard (yearly)	Yes			Yes			Yes			Yes			Yes		
Number of person-years	65,008			65,008			65,008			65,008			65,008		
Number of observations	13,948			13,948			13,948			13,948			13,948		
AIC	41,596			40,264			40,264			39,903			39,904		
BIC	41,760			40,609			40,627			40,294			40,304		

Hazard ratios based on Eq. (3) are reported and standard errors clustered at the individual level are in parentheses

*p < 0.05; **p < 0.01; ***p < 0.001 (two-tailed)

These results show that the reason for migration and individuals’ income are strongly related to the risk of return migration. The results further indicate that the reason for migration and trajectories at the destination are interdependent, also after the year of arrival in Sweden. Additional results that are based on stratified analyses by the reason for migration show that income plays an important role in all the migrant groups’ decisions to return migrate (see Table S3 in the Supplement). However, differences in return migration risks across income quartiles are less marked among labour migrants than in the other three groups.

### Circular Migration

As a final exercise, we analyse circular migration. First, we assess seasonal patterns in circular migration among labour, student, tied migrants and migrants in the residual category. Second, we present survival curves for the risk of circular migration, i.e. second immigration and second return migration, respectively. For the second immigration, the risk set comprises migrants who return migrated to Finland (N = 8145). For the second return migration, the risk set comprises migrants who immigrated to Sweden for a second time (N = 1479). Right-censoring is handled as described earlier.

**Fig. 4:**
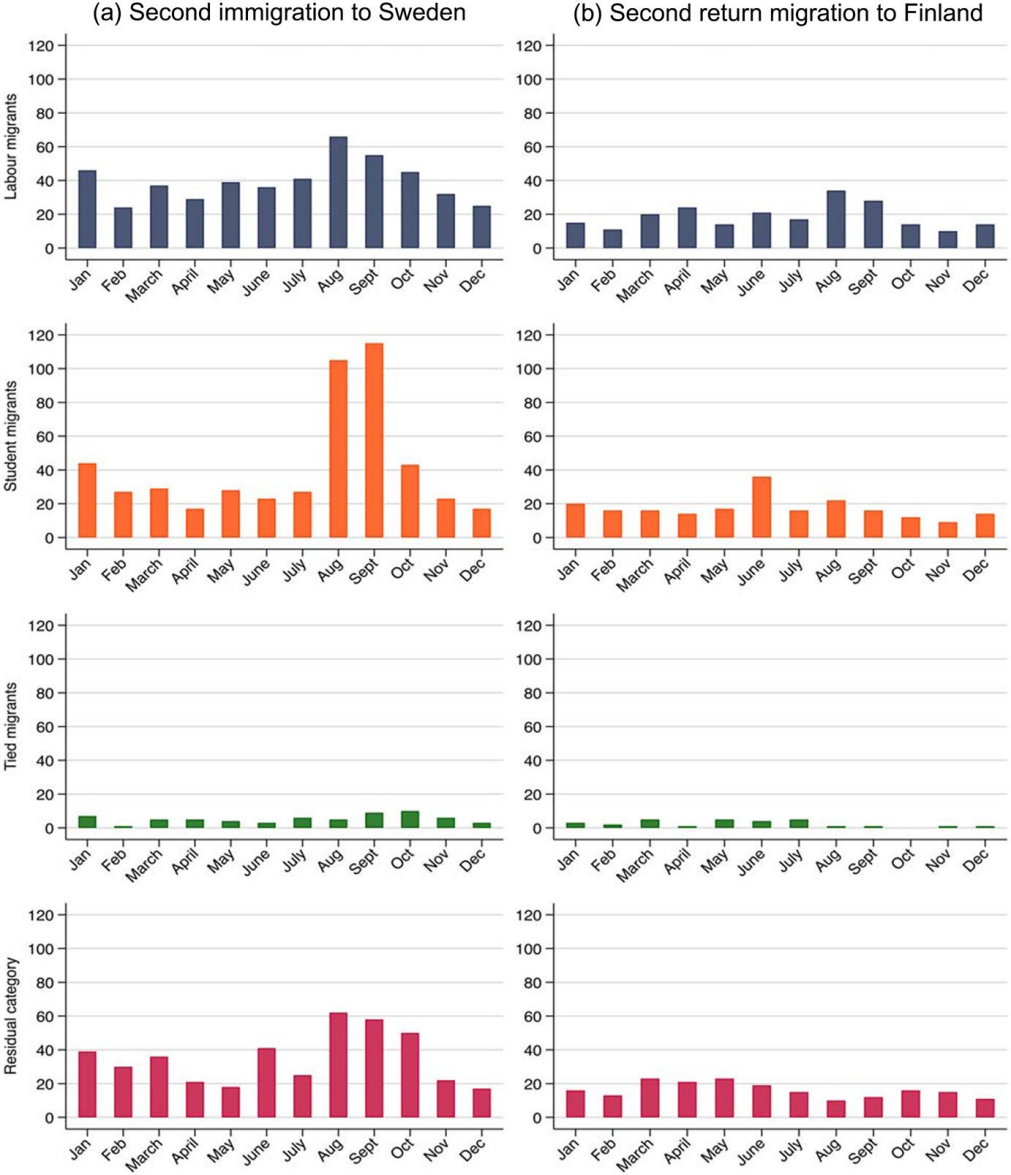
Number of circular migrants (making a second immigration to Sweden or a second return migration to Finland) by month of migration and reason for migration. *Note* The x-axis refers to the number of individuals

Figure 4 shows that seasonal patterns for circular migration are similar to those observed for immigration and return migration. Student migrants are more prone to immigrate for a second time in August and September and to return for a second time in June, which is in line with the pattern observed for first immigration and first return migration (see Fig. 1). Due to the small number of observations, it is difficult to distinguish seasonal patterns for the other migrant groups.

**Fig. 5:**
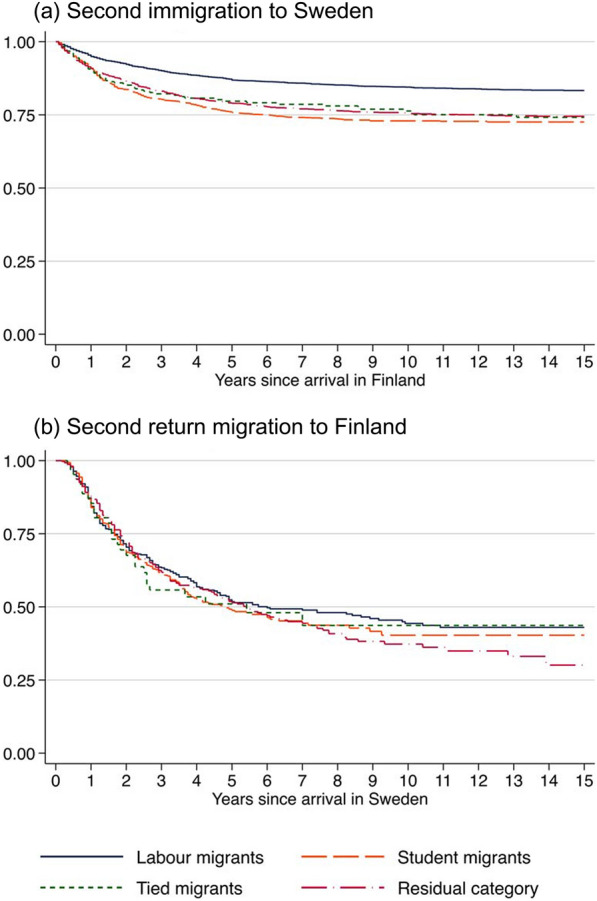
Survival estimates for circular migration (making a second immigration to Sweden or a second return migration to Finland) by the reason for migration. *Note* Corresponding survival probabilities are provided in Table S5 in the Supplement

When it comes to the likelihood of engaging in circular migration, student migrants are the most likely group to immigrate for a second time, followed by tied migrants and migrants in the residual category (see Fig. 5).^6^ Labour migrants are considerably less likely to immigrate for a second time, probably because they have returned due to low income. For the second return migration, differences between the groups are small.

## Conclusion

In settings of free mobility, the threshold to moving is low. This allows for a wider range of migration motives, settlement plans and integration patterns (Burrell, 2010; Kleinepier et al., 2015; Luthra et al., 2018). Migration as well as income and family trajectories is generally difficult to disentangle, as all three occur during early adulthood and influence one another (King, 2002; Sirniö et al., 2017; Vidal et al., 2020). This is arguably yet more complex in settings of free mobility where individuals have a greater set of options available to them. Studying these three trajectories in an accentuated form has allowed us to gain insight into some of the dynamics underlying migration and integration.

Using linked Finnish and Swedish register data, we studied how the reason for migration and trajectories at the destination relates to return migration. Longitudinal individual-level data that link migrants’ experiences in the origin and destination country allowed us to approximate the reason for migration in register data. The data further provided us with a 15-year follow-up period to assess migrants’ short- as well as longer-term outcomes. This set-up enabled us not only to show how the processes are connected empirically, but also to bridge different parts of the literature. Beyond contributing to the literature on intra-European migration by analysing the Finnish-Swedish migration context, we incorporated a part of the literature that focuses on the relationship between integration and return migration.

Our first research aim was to approximate the reason for migration. In relation to this, labour migrants were found to account for a large proportion, or 42%, of the migrant population, in spite of presumably low economic incentives to move during the study period when compared to prior decades. In the literature, student migrants have been considered to be a growing group who are difficult to identify in administrative data (Kelo et al., 2006; King, 2018; Wit et al., 2013). We found that student migrants constitute nearly a third of all migrants analysed (29%).

Our second research aim was to assess income and family trajectories across migration motives. Results revealed that labour migrants’ income was initially higher than that of the other migrant groups. This may be due to the fact that labour migrants move for work and with the aim of increasing returns to their skills, as argued by the *human capital model* (Harris & Todaro, 1970; Maskileyson et al., 2021; Sjaastad, 1962). However, over time in the destination country, economic differences between the groups decreased. In particular, a steep earnings curve was observed among student migrants. This appeared to be driven by qualifications gained in the destination country, which may be valued by local employers. Tied migrants were more likely to enter the country with children or a spouse, whereas student migrants waited longer to form a family than the other migrant groups. Similar patterns of family formation have been observed among Polish migrants in the Netherlands (Kleinepier et al., 2015). It may be that student migrants wait longer to form a family as they tend to be younger, but it could also be that they move to gain new experiences rather than to settle down, as hypothesised by *global youth mobility cultures* (Findlay et al., 2017; King & Ruiz‐Gelices, 2003). These findings contribute to prior studies on integration by assessing diversity across migrants using pre- and post-migration information from the origin and destination country and studying how different migrant groups fare over time in the destination country.

Our third research aim was to study how return migration risks are shaped by the migration motive as well as income trajectories and trajectories of family formation. We found that, at similar levels of income, labour and student migrants were more likely to return migrate than the other groups. Migrants’ income and family formation in the destination country were negatively related to the risk to return, which is in line with prior evidence on the Finnish–Swedish context (Saarela & Scott, 2017; Weber & Saarela, 2019), and other contexts (Constant & Zimmermann, 2011, 2012; Kleinepier et al., 2015). Our results indicate that the initial migration motive and migrants’ situation in the destination country do not operate independently, highlighting the importance of studying these processes within the same empirical framework.

Our fourth research aim was to study how the reason for migration relates to seasonal patterns and the likelihood of circular migration. We found that seasonal patterns for circular migration were highly similar to those observed for immigration and return migration. Student migrants were the most likely group to immigrate for a second time. This result is in line with previous findings on continued mobility among students who participated in the Erasmus programme (Findlay et al., 2017; King & Ruiz‐Gelices, 2003; Parey & Waldinger, 2011). This may be in part due to the social network and language skills that student migrants acquire during their studies abroad (Zwysen, 2019).

Even though we have studied migration and integration between two neighbouring countries with strong historical ties, we think there are opportunities for external validity. Arguably, the more general mechanisms analysed span across migration contexts. For instance, migrants are often young adults who simultaneously try to find a place to live, seek employment and start their family. That said, all migration contexts are subject to specific contextual factors, which implies that one should always be cautious about generalising results. In order to disentangle context-specific factors and general mechanisms, it is important to study how similar processes manifest in different contexts. While previous studies have focused on migration flows from East to West Europe (Castro-Martín & Cortina, 2015), this study has analysed the Finnish-Swedish migration context. This setting has provided us with longitudinal individual-level data, which allowed us to connect migrants’ experiences on both sides of the border. Furthermore, the common Nordic labour market was a predecessor for the idea of free movement in the EU and may therefore point to potential developments of mobility in the Schengen area.

There are at least two major limitations of the study that need to be stressed. First, we approximated the reason for migration using a rather traditional typology. It may therefore be that we do not fully capture the breadth of migration motives, and the motives may certainly change over time. It may also be that individuals have multiple reasons for migration. Tied migrants, in particular, are difficult to classify using one reason for migration. For instance, individuals may primarily move to be with their partner, but are classified as labour or student migrants if they work or study in Sweden. Still, we aimed at establishing a framework that allows us to capture heterogeneity across migrants regarding trajectories at the destination and subsequent moves in both directions using linked register data. Second, we used individual-level information and thereby only proxied social contacts. We could not observe family members explicitly, nor did we have information on their personal characteristics. Including such information in similar data sets will be important for future research in the field.

Nonetheless, the findings have a number of implications. Using longitudinal data and observing migrants over a 15-year period provided insight into detailed trajectories. These revealed considerable heterogeneity within the migrant population, especially when it comes to how young migrants structure their lives in the destination and/or origin country. This implies that it is important for policy makers to consider diversity across migrants, even from the same country of origin, when designing integration policies. Our findings also indicated that information from both the origin and destination country is valuable for gaining a more complete picture of the heterogeneity in integration and migration risks across different migrant groups. In this way, it will be important to incorporate data from both the origin and destination country more prominently in empirical and theoretical research.

## Data Availability

The analysis presented in the study is based on linked Finnish and Swedish register data. The data are considered sensitive and were made available to the project on the condition that there cannot be further distribution of the data. For access to the underlying data, interested researchers are asked to contact the national statistics agencies of the respective countries.

## Footnotes

1 The permission number from Statistics Sweden is 8547689/181453 and from Statistics Finland TK-52-215-11.

2 Models do not control for birth cohorts due to high correlation between year of immigration and birth cohort (Pearson’s r is 0.83). In additional analyses, we control for age at immigration and birth cohorts instead of the year of immigration (see Figs. S1–S2 and Table S4 in the Supplement).

3 Adjusted predictions are estimated using the Stata command: margins, at(year_since_mig = (1(1)15) reason_mig = (1 2 3 4)) atmeans post (Williams, 2012).

4 We model parenthood and marriage together, as results from separate analyses reveal similar patterns (see Figures S3–S4 in the Supplement).

5 Adjusted predictions are estimated using the Stata command: margins, at(year_since_mig = (1(1)15) reason_mig = (1 2 3 4)) atmeans post (Williams, 2012).

6 Survival probabilities are provided in Table S5 in the Supplement.

## Code Availability

Considering the nature of the data, the code has not been submitted but is available from the authors upon request.
